# The biomechanical effect of fibular strut grafts on humeral surgical neck fractures with lateral wall comminution

**DOI:** 10.1038/s41598-023-30935-y

**Published:** 2023-03-06

**Authors:** Hsien-Hao Chang, Joon-Ryul Lim, Kil-Han Lee, Haemosu An, Tae-Hwan Yoon, Yong-Min Chun

**Affiliations:** 1 Department of Orthopaedic Surgery, Arthroscopy and Joint Research Institute, Severance Hospital, Yonsei University College of Medicine, 50-1 Yonsei-ro, Sinchon-dong, Seodaemun-gu, Seoul, 03722 Korea; 2 Surgical Anatomy Education Centre, Yonsei University College of Medicine, Seoul, Korea

**Keywords:** Musculoskeletal system, Biomedical engineering

## Abstract

No studies have evaluated the effect of fibular strut augmentation on the stability of locking plate fixation for osteoporotic proximal humeral fractures with lateral wall comminution. The purpose of this study was to evaluate the stability of locking plate fixation with a fibular strut graft compared with locking plate alone in an osteoporotic two-part surgical neck fracture model with lateral cortex comminution. Ten paired fresh-frozen cadaveric humeri were randomly allocated into two groups, either the locking plate alone (LP group) or locking plate with fibular strut graft augmentation (LPFSG group), with an equal number of right and left osteoporotic surgical neck fractures with lateral wall comminution of the greater tuberosity. Varus, internal/external torsion, and axial compression stiffness as well as single load to failure were measured in plate-bone constructs, and the LPFSG group showed significantly greater values in all metrics. In conclusion, this biomechanical study shows that fibular strut augmentation significantly enhances varus stiffness, internal torsion stiffness, external torsion stiffness, and maximum failure load of a construct compared to locking plate fixation alone in proximal humeral fractures with lateral wall comminution.

## Introduction

Proximal humeral fractures are one of the most common fractures in the elderly, and the incidence of proximal humeral fractures has increased with longer life expectancy^1–3^. Approximately 70% of all proximal humeral fractures occur in patients aged 60 and older^4^. Most cases can be treated conservatively; however, surgical treatment is favored over conservative treatment if the fracture is unstable and displaced^5^.

Stable fixation followed by adequate rehabilitation has shown satisfactory outcomes in proximal humeral fractures, and among the many fixation methods, locking plate techniques have shown good results in patients with compromised bone quality^6,7^. In addition, calcar screws and strut bone grafts have been associated with enhanced mechanical stability against varus collapse and satisfactory surgical outcomes in proximal humeral fractures with medial metaphyseal comminution or poor bone quality related to osteoporosis^8,9^.

Typically, solid fixation can be achieved through the use of plate and screw fixation of the near and far cortex^10^. However, in the proximal humerus fracture, fixation is dependent on the cancellous bone within the humeral head and the cortical bone of the lateral wall of the greater tuberosity, which are frequently involved in proximal humerus fracture. The involvement of the greater tuberosity (GT) is known to be common with its incidence up to 20% among all types of proximal humerus fractures^11^. Furthermore, the fixation plate is commonly placed lateral wall of the GT^12,13^. Considering that proximal humeral fractures occur in the elderly, stability of fixation may be significantly compromised by lack of lateral wall integrity of the greater tuberosity, especially in cases of low bone mass in the humeral head^4^. Although it is well known that fibular strut grafts have enhanced the results of locking plate fixation in unstable osteoporotic proximal humerus fractures with medial wall comminution^8,14^, no studies have evaluated the effect of fibular strut augmentation on the stability of locking plate fixation for osteoporotic proximal humeral fractures with lateral wall comminution.

In this study, we aim to evaluate the stability of locking plate fixation with a fibular strut graft compared with locking plate alone in an osteoporotic two-part surgical neck fracture model with lateral cortex comminution. We hypothesized that augmentation with a fibular strut graft will enhance the biomechanical stability of locking plate fixation in proximal humeral fractures with lateral wall comminution.

## Methods

### Specimen preparation

Ten pairs of fresh-frozen cadaveric humeri without any gross deformities or a history of injury or operation were used in this study. All specimens were stored frozen at − 20 °C and thawed at room temperature 24 h before use. All soft tissue of the humerus were thoroughly removed before use.

**Figure 1:**
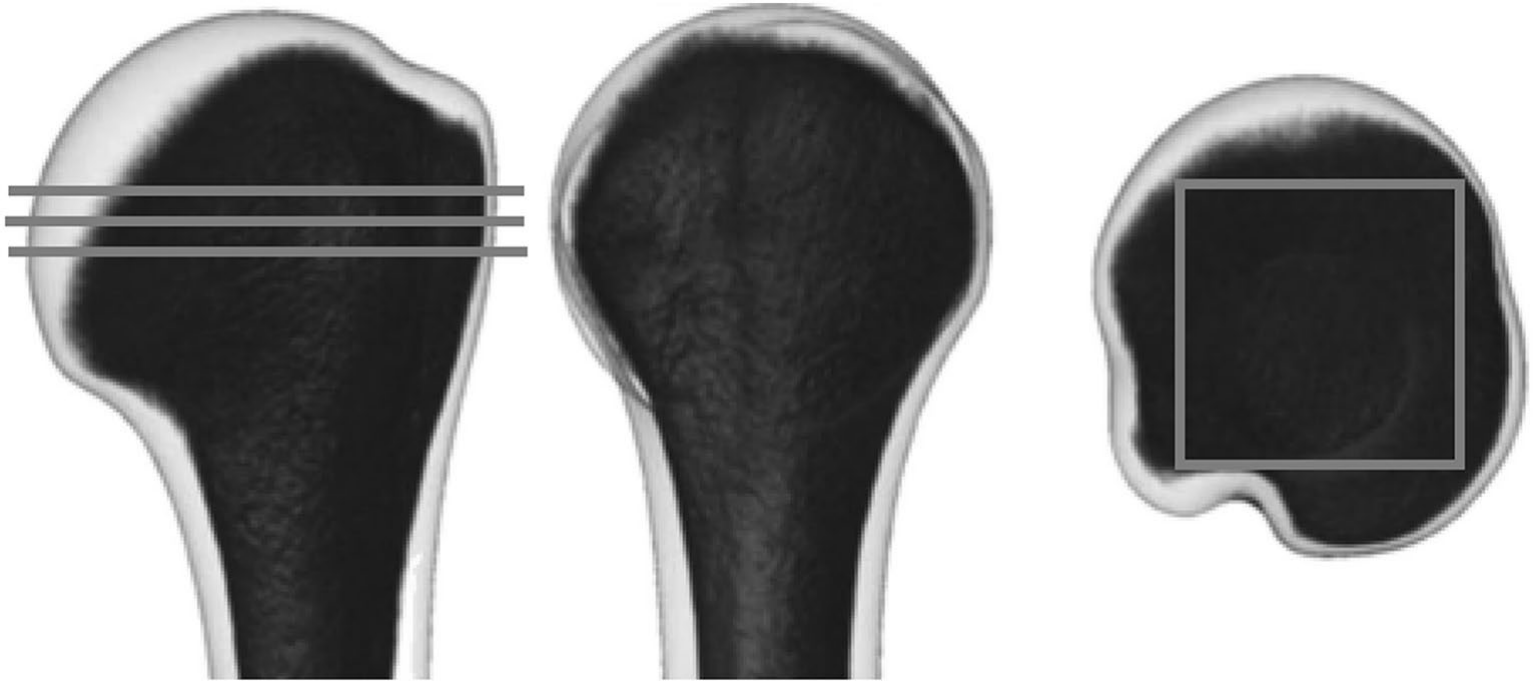
A schematic figure for measuring BMD of the humeral head.

Quantitative computed tomography was used to measure bone mineral density (BMD) of each humeral head. BMD was evaluated at the greatest transverse diameter of the humeral head along three parallel sections separated by 1.5-mm distance^15^. The square region of interest (ROI) was positioned over the bone slice so that every edge of the square could reach the subcortical shell of the humeral head. The mean BMD of the three slices was used^15^. (Fig. 1).

**Figure 2:**
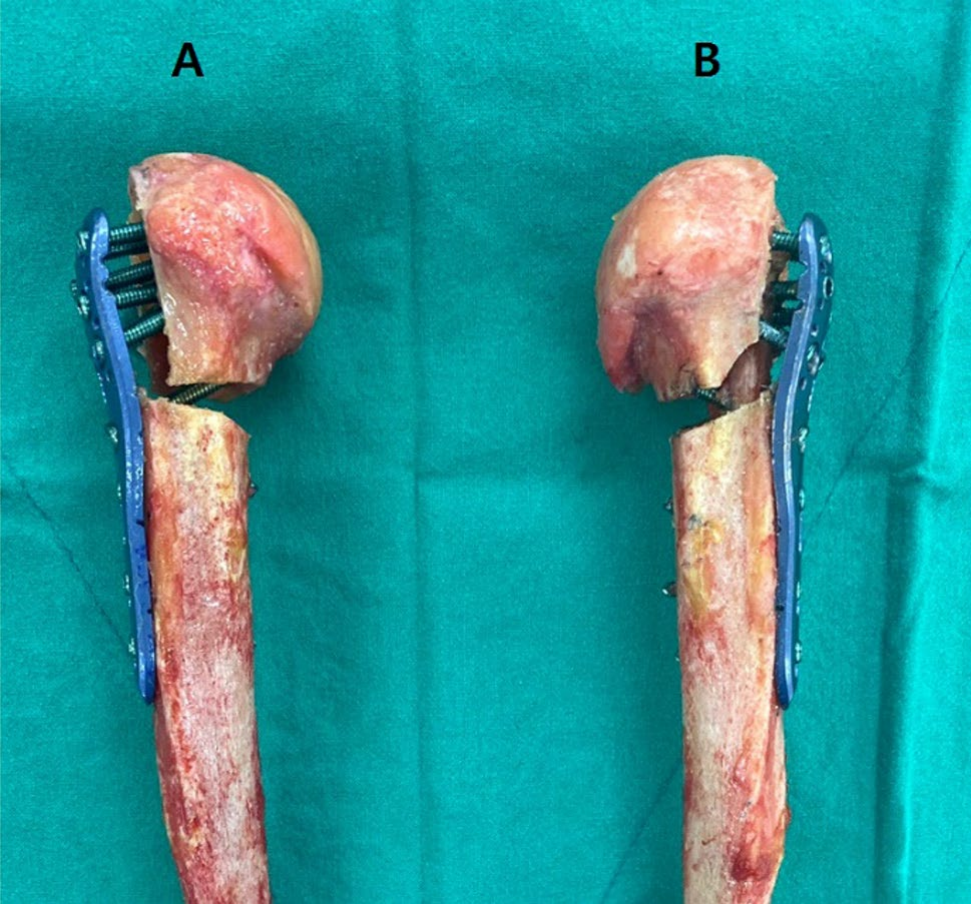
A two-part surgical neck fracture with a comminuted lateral wall of the greater tuberosity fixed with a locking plate alone (**A**) and a locking plate with fibular strut graft augmentation (**B**).

**Figure 3:**
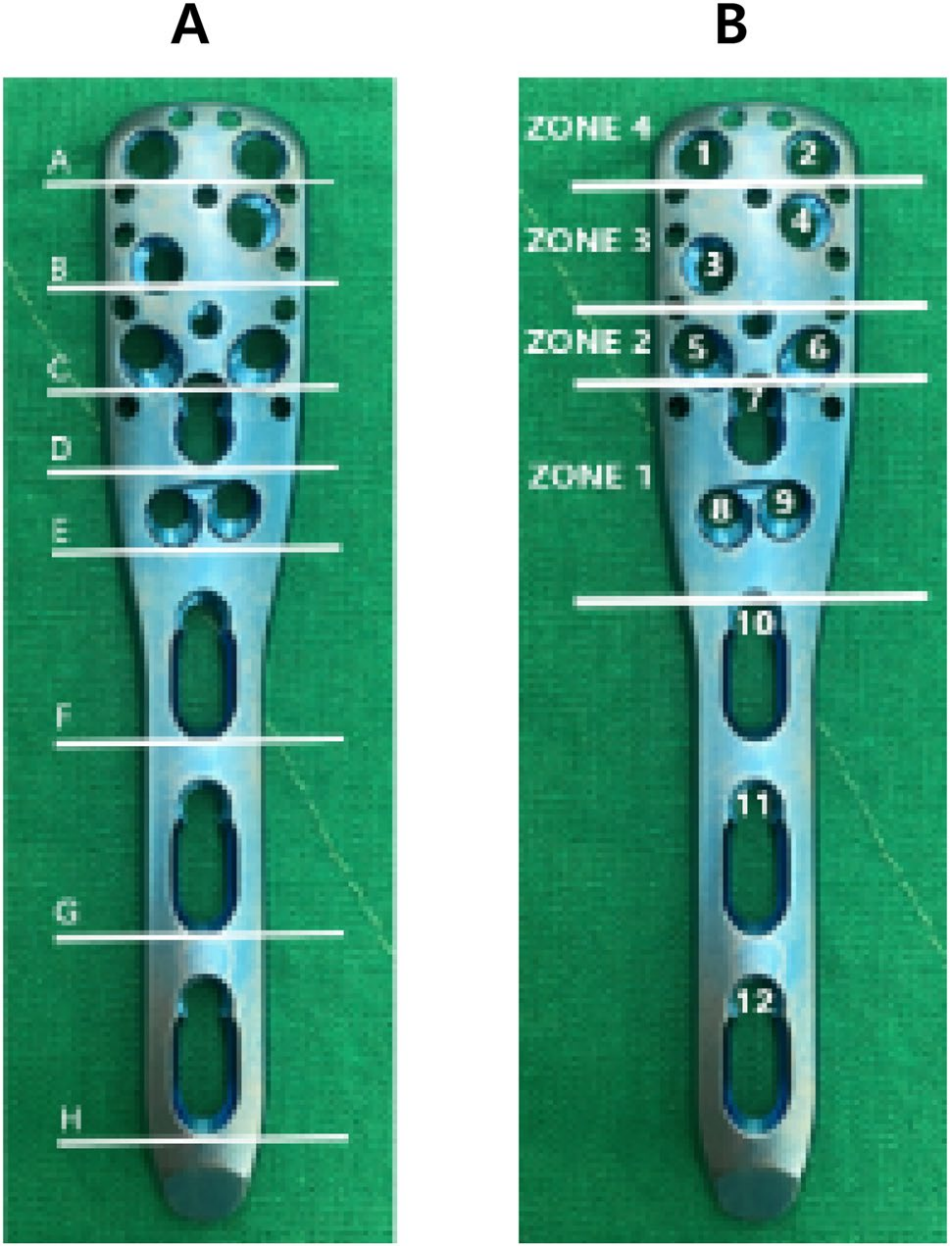
(**A**) A PHILOS humerus plate with 9 proximal screw holes in Sections A-E for locking screws, 10 proximal suture holes to help maintain fracture reduction, and 3 distal screw holes. (**B**) Screws were categorized into several zones based on position^20^.

A two-part surgical neck fracture with a comminuted lateral wall of the greater tuberosity model was created by performing osteotomy at the following points using a microsagittal saw (Fig. 2): (1) a 5-mm wedge shaped gap one centimeter distal to the most inferior portion of the articular cartilage on the humeral head perpendicular to the humeral shaft^16–19^ and (2) a 5-mm-thick segment posterior to the bicipital groove corresponding to zones 2, 3, and 4 of the applied Proximal Humerus Internal Locking System (PHILOS) plate (Fig. 3), leaving two millimeters of the tip of the greater tuberosity intact to serve as a reference for locking plate application^13,20,21^.

Block randomization was used to allocate the paired specimens (left and right side) into two groups: (1) locking plate alone (LP group) and (2) locking plate with fibular strut graft augmentation (LPFSG group). Each group was assigned an equal number of right and left humeri. All specimens were fixed with the same locking plate (PHILOS; Synthes, Paoli, Pennsylvania) using 12 screw holes (three distal and nine proximal) and the standard technique (Fig. 3). Plates were fixed on the lateral wall of the proximal humerus using six locking screws on the most proximal screw holes (Sections A, B, and C) and three screws on the shaft (Sections F, G, and H). Two additional screws were inserted on Section E (Fig. 3) for medial calcar support. Specimens in the locking plate with fibular strut graft augmentation group were fixed with an additional intramedullary fibular strut graft. An 80 mm segment from the ipsilateral fibular diaphysis of the cadaver was inserted into the medullary cavity of the diaphysis and humeral head. Then, 5 cm of the graft was impacted into the diaphysis and stabilized with three locking screws.

The humeral shaft was cut transversely at 16.5 cm from the upper margin of the wedge resection that is perpendicular to the anatomical axis of the humeral shaft and secured in a 7-cm-long tube with unsaturated polyester resin (EC‐304, Aekyung Chemical Co.)^22,23^. The humeral head was placed in unsaturated polyester resin up until 2 cm from the proximal wedge-shaped cut at the surgical neck and mounted to a customized jig.

### Biomechanical testing

Measurement variables for biomechanical testing included axial compression, varus bending, torsional stiffness, and one single load to failure of varus bending, which commonly used in mechanical testing^24^. Biomechanical axial compression and varus bending and single load to failure tests were conducted using an electrohydraulic materials test system (model 3366; Instron, Norwood, Massachusetts), and rotational stiffness was measured using a torsional stiffness tester (DPTST; DYPHI).

**Figure 4:**
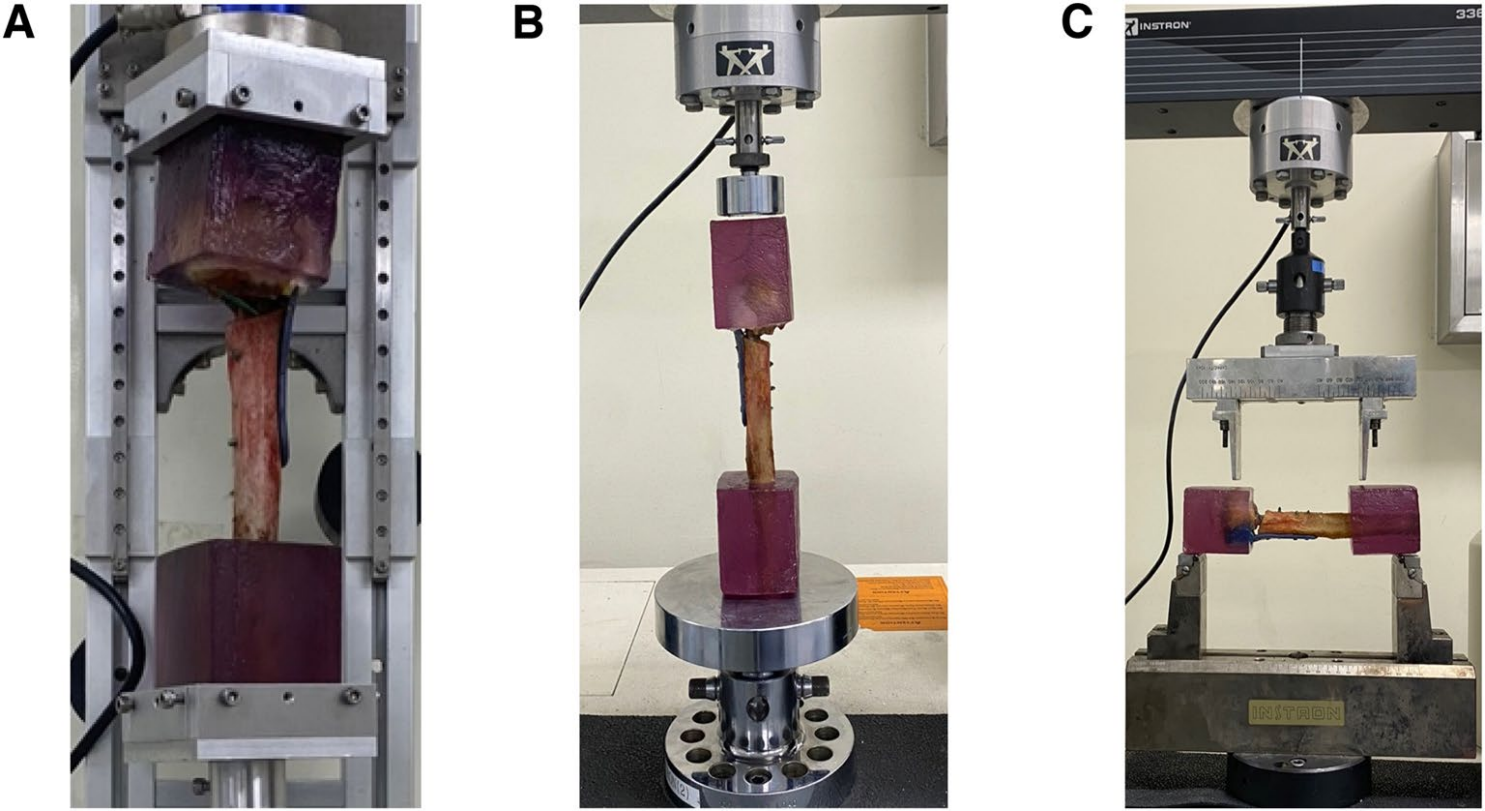
(**A**) Test for rotational stiffness using a torsional stiffness tester (DPTST; DYPHI). (**B**) Test for axial compression using an electrohydraulic materials test system (model 3366; Instron, Norwood, Massachusetts). (**C**) Test for four-point bending using an electrohydraulic materials test system (model 3366; Instron, Norwood, Massachusetts).

**Figure 5:**
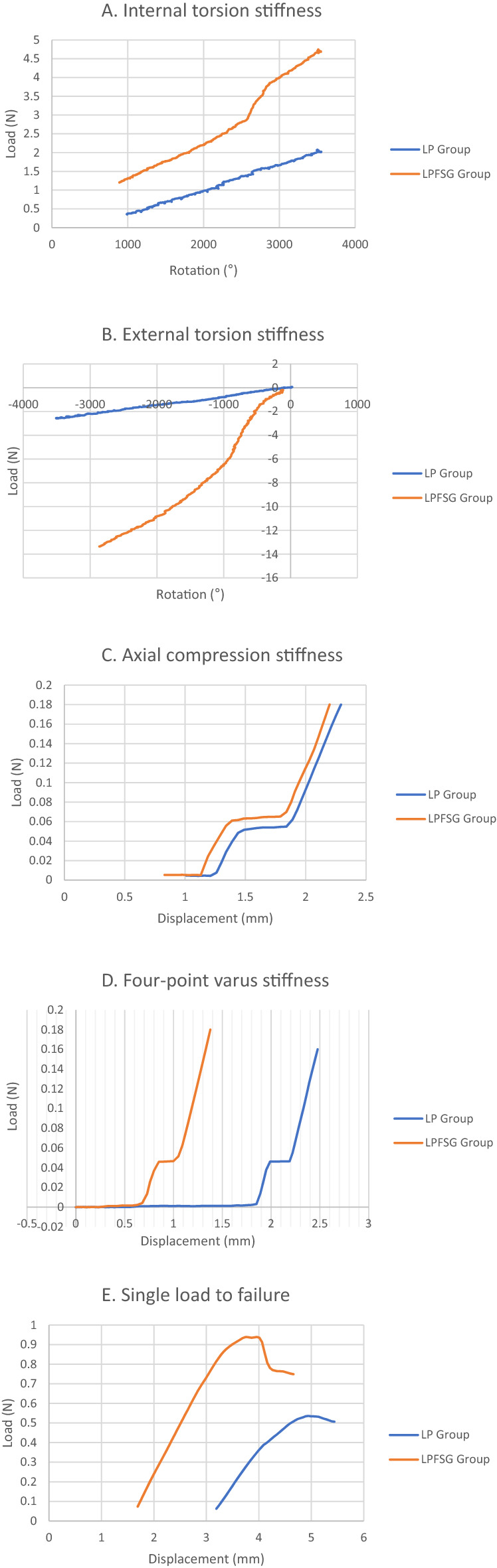
Load displacement curve of (**A**) Internal torsion stiffness, (**B**) External torsion stiffness, (**C**) Axial compression stiffness, (**D**) Four-point varus stiffness, and (**E**) Single load to failure.

First of all, quasi-static internal and external torsional tests were performed, with rotating the humeral head at 0.2Nm torque per second + 3.5 Nm and − 3.5 Nm for internal and external torsional stiffness, respectively (Fig. 4A). Secondly, axial compression test was performed with an axial force up to 200N at a rate of 0.1 mm per second (Fig. 4B). The four-point varus bending stiffness test was performed using an electrohydraulic materials test system setup (Fig. 4C), with a supporting span of 21 cm, and a loading span of 13 cm, with 3.5Nm at 0.1 mm per second. The stiffness was determined by calculating the slope of the linear region in the force/displacement graph at the fifth cycle (Fig. 5A–D). Finally, the single load to failure test was measured in the four-point varus test setup, until a sudden change occurred due to loss of fixation in the force/displacement curve^25^ (Fig. 5E).

### Statistical analysis

Sample size calculation for comparison between two groups requires estimates of treatment effect^26,27^; however, because there were no previous studies in the literature, we performed a pilot study using three paired humeri (six specimens). Sample size was calculated using the varus stiffness values, and the mean ± standard deviation values for the LP group and LPFSG group were 338.69 ± 158.54 N/mm and 493.44 ± 171.91 N/mm, respectively. Accordingly, 10 specimens were required to achieve a power of 80% at an α level of 0.05.

Due to the small sample size, we used the non-parametric Wilcoxon signed-rank test to compare the differences in mechanical testing values between the paired samples. Statistical significance was set at p < 0.05, and statistical analyses were performed using IBM SPSS Statistics for Windows (version 25.0; IBM Corp.).

### Ethics statement

This study was approved by the Institutional Review Board of Yonsei University Health System, Severance Hospital (4-2022-0989). All experiments were performed in accordance with relevant guidelines and regulations. The informed consent was obtained from the donors or their legal guardians.

## Results

The mean BMD for the LP group was 32.10 mg/cm^3^ and 33.69 mg/cm^3^ for the LPFSG group. The two groups did not show a significant difference in BMD (p = 0.139). The mean varus stiffness, mean internal torsion stiffness, mean external torsion stiffness, mean axial compression stiffness, and single load to failure values were 265.32 N/mm, 2.34 N/deg, 1.72 N/deg, 320.92 N/mm, and 199.50 N in the LP group, respectively, and 417.65 N/mm, 0.72 N/deg, 0.68 N/deg, 384.55 N/mm, and 455.45 N in the LPFSG group. The LPFSG group showed significantly greater values in all biomechanical metrics excluding axial compression stiffness (Table 1).

**Table 1:** Comparative results of bone marrow density (BMD), stiffness, and single load to failure between the LP group and LPFSG group.

	LP Group (range)	LPFSG Group (range)	p-value
BMD (mg/cm^3^)	32.10 ± 14.97 (13.41–52.92)	33.69 ± 15.18 (12.24–58.92)	0.139
Varus stiffness (N/mm)	265.32 ± 146.71 (61.85–447.04)	417.65 ± 116.27 (258.14–623.21)	0.014
Internal torsion stiffness (N/deg)	2.34 ± 0.86 (0.95–3.45)	0.72 ± 0.42 (0.21–1.64)	0.003
External torsion stiffness (N/deg)	1.72 ± 0.93 (0.98–3.51)	0.68 ± 0.25 (0.35–1.04)	0.003
Axial compression stiffness (N/mm)	320.92 ± 182.16 (92.99–590.26)	384.55 ± 127.95 (109.12–513.60)	0.223
Single load to failure (N)	199.50 ± 119.76 (54.00–365.37)	455.45 ± 416.53 (196.80–1609.80)	0.011

## Discussion

In the osteoporotic proximal humeral fracture with lateral wall comminution model of this study, we compared the stability of locking plate alone versus locking plate fixation with fibular strut graft augmentation. As we hypothesized, augmentation with a fibular strut graft significantly enhanced the biomechanical stability of locking plate fixation in proximal humeral fractures with lateral wall comminution in terms of varus stiffness, internal and external torsion stiffness compared with that of lateral wall comminution alone.

Due to comminution and a low bone mass within the humeral head in elderly patients, fixation failure is frequent despite the developments in fixation materials and techniques, including a locking plate and screw system. Particularly in surgical neck fractures with medial cortex comminution, the importance of medial calcar support screw fixation has been emphasized in many biomechanical and clinical studies^28,29^. On the contrary, although many proximal humerus fractures involve the lateral wall of the GT where the plate is applied, it has not been highlighted. Supposedly, this is because non-displaced lateral wall fractures of the GT with comminution are only identified on computed tomography (CT) and can be easily missed on plain x-ray.

Once the lateral wall is involved, application of the locking screw and plate system on the fractured lateral wall seems to be difficult to provide stable fixation, especially when the quality of humeral head bone is poor due to osteoporosis. Naturally, postoperative rehabilitation including ROM exercise is very limited due to concern about fixation failure and morbidities in elderly patients resulting from subsequent re-operation.

The endosteal fibular allogenous strut bone graft technique was introduced to improve fixation stability in cases of proximal humerus fracture with medial cortex comminution with low bone mass^30^. However, we thought that it would be helpful in cases of concomitant lateral wall fracture with osteoporosis; therefore, an unstable medial column model with osteoporosis was created with elderly cadaver specimens as in a previous study^29^, and unstable lateral column components were added. If loss of lateral wall integrity and low bone mass within the humeral head are present, the stability of the proximal fragment will not be sufficient, even with the medial calcar screw. Recently, Jang et al. published a study comparing locking plate with medial support screw and locking plate with intramedullary fibular graft fixation in varus collapsed proximal humerus fracture models^31^. Despite several studies reporting the advantages of the medial support screw, the fibular strut graft showed significantly better biomechanical stability than the medial support screw.

As we hypothesized, the use of fibular strut bone graft significantly improved fixation stability compared to the LP group in the osteoporotic proximal humerus fracture model with lateral wall comminution. Thus, the presence of lateral wall comminution of the greater tuberosity should be identified in preoperative planning, and strut bone augmentation should be considered for sold fixation in osteoporotic proximal humerus fractures.

To the best of our knowledge, this is the first biomechanical study comparing the stability of locking plate alone versus locking plate fixation with fibular strut graft augmentation in a proximal humeral fracture model with lateral wall comminution. However, our study has several limitations. First, as in most other cadaver studies, this was time-zero research. In studies using cadavers, additional bracing typically yields better fixation stability. However, it is well known that the strut bone blocking the medullary canal will become an obstacle in conversion to arthroplasty. Therefore, the use of strut bone augmentation should be carefully determined in preoperative planning and surgical field assessment on a case-by-case basis. Second, lateral wall comminution in an osteoporotic proximal humerus fracture model had no conventional reference. Although we tried our best to simulate the most similar model based on the clinical data experienced in our institute, we would not have the same specimens represented in the actual lateral wall comminution models. The degree of osteotomy was determined by referring to the existing surgical neck comminuted fracture model.

In conclusion, this biomechanical study shows that fibular strut augmentation significantly enhances the varus stiffness, internal torsion stiffness, external torsion stiffness, and maximum failure load of a construct compared to locking plate fixation alone in proximal humeral fracture models with lateral wall comminution.

## Data Availability

The datasets analysed during the current study are available from the corresponding author on reasonable request.
