# Author Correction: The biomechanical effect of fibular strut grafts on humeral surgical neck fractures with lateral wall comminution

**DOI:** 10.1038/s41598-023-32259-3

**Published:** 2023-03-29

**Authors:** Hsien-Hao Chang, Joon-Ryul Lim, Kil-Han Lee, Haemosu An, Tae-Hwan Yoon, Yong-Min Chun

**Affiliations:** 1 Department of Orthopaedic Surgery, Arthroscopy and Joint Research Institute, Severance Hospital, Yonsei University College of Medicine, 50-1 Yonsei-ro, Sinchon-dong, Seodaemun-gu, Seoul, 03722 Korea; 2 Surgical Anatomy Education Centre, Yonsei University College of Medicine, Seoul, Korea

Correction to: *Scientific Reports* 10.1038/s41598-023-30935-y, published online 06 March 2023

The original version of this Article omitted an affiliation for all authors. The correct affiliations are listed below.

Department of Orthopaedic Surgery, Arthroscopy and Joint Research Institute, Severance Hospital, Yonsei University College of Medicine, 50-1 Yonsei-ro, Sinchon-dong, Seodaemun-gu, Seoul, 03722, Korea.

Surgical Anatomy Education Centre, Yonsei University College of Medicine, Seoul, Korea.

Additionally, the Acknowledgments section was omitted. The Acknowledgments section now reads:

“We thank JH Kim, JH Bang, and T-J Ha from the Surgical Anatomy Education Center at the Yonsei University College of Medicine, Seoul, for technical support.”

The original Article has been corrected.

